# Disruption of cholesterol homeostasis by antidepressants induces immunogenic cell death

**DOI:** 10.1080/2162402X.2025.2531113

**Published:** 2025-07-10

**Authors:** Karla Alvarez-Valadez, Jonathan G. Pol, Guido Kroemer, Mojgan Djavaheri-Mergny

**Affiliations:** a Centre de Recherche des Cordeliers, Inserm UMRS 1138, Sorbonne Université, Université de Paris Cité, Équipe labellisée par la Ligue contre le Cancer, Institut Universitaire de France, Paris, France; b Université Paris-Saclay, INSERM US23/CNRS UAR 3655, Metabolomics and Cell Biology Platforms, Institut Gustave Roussy, Villejuif, France; c Department of Biology, Institut du Cancer Paris CARPEM, Hôpital Européen Georges Pompidou, AP-HP, Paris, France

**Keywords:** Antitumor immunity, cancer, cholesterol transport, lysosome, serotonin reuptake inhibitors, TFEB

## Abstract

Sertraline and indatraline are two antidepressants that function as serotonin reuptake inhibitors and have demonstrated promising anticancer potential, although their precise mechanisms of action remain unclear. Both compounds trigger cholesterol accumulation within lysosomes followed by lysosomal membrane permeabilization, ultimately leading to the activation of immunogenic cell death (ICD). This, in turn, triggers a T cell-mediated adaptive immune response that facilitates significant tumor control.

Lysosomes play pivotal roles in cell survival, growth, metabolism, and immune responses.^1^ In cancer cells, the disruption of lysosomal function and integrity can activate several cell death pathways, offering an effective strategy to combat malignant diseases. Notably, due to changes in their lipid and protein composition, the lysosomes of cancer cells are particularly vulnerable to treatments that induce lysosomal membrane permeabilization (LMP).^2^ Moreover, LMP can trigger cell death in cancer cells that lack components of the apoptotic machinery, thus providing a promising strategy to overcome resistance to certain therapies. Cationic amphiphilic drugs (CAD) which have hydrophobic and cationic properties, have garnered attention for their capacity to induce lysosomal damage and suppress tumor growth across various preclinical models.^3^

Immunogenic cell death (ICD) encompasses regulated cell death modalities capable of eliciting an adaptive immune response against cancer cells.^4^ ICD is characterized by the emission of danger signals in the form of damage-associated molecular patterns (DAMPs) from dying tumor cells. Such dangers signals include the exposure of calreticulin (CALR) on the cell surface, the secretion of ATP, and the release of high mobility group box 1 (HMGB1), which collectively promote tumor-specific immune responses. ICD can be triggered by various stressors including reticulum endoplasmic stress and the production of reactive oxygen species.^5,6^ While chemotherapy-induced ICD has been widely studied,^7^ whether LMP-inducing agents can initiate ICD in cancer cells remains largely unexplored.

Recently, we reported that sertraline and indatraline, two antidepressants that function as serotonin reuptake inhibitors, act as CAD. Both compounds were capable of inducing LMP and triggering key hallmarks of ICD in a variety of cancer cell lines.^8^ Sertraline and indatraline displayed notable cytotoxicity in various cancer cell lines and provoked the exposure of CALR and the release of both ATP and HMGB1, thereby establishing their immunogenic potential. This was further substantiated *in vivo* through vaccination-rechallenge and therapeutic experiments in immunocompetent mice. In both prophylactic and established sarcoma models, a single administration of either drug led to significant tumor growth inhibition. CD4 or CD8 T cell depletion abrogated these therapeutic effects, confirming a T cell-dependent response (Figure 1). Notably, immunocompetent mice that were protected from tumor development following injection with syngeneic sarcoma cells treated *in vitro* with sertraline or indatraline remained tumor-free when rechallenged with live sarcoma cells, indicating that they developed durable immune memory. Consistent with our findings in sarcoma models, the role of T cells in sertraline-mediated tumor growth suppression was recently confirmed in other tumors models as well.^9^ Further studies are needed to determine whether the immunostimulatory effects of CAD antidepressants also involve tumor cell-extrinsic mechanisms, such as direct modulation of immune cells, and whether their immunogenic potential could convert immunologically cold tumors into hot, inflamed microenvironments, thereby enhancing responsiveness to broadly effective immune checkpoint inhibitors.

**Figure 1.**
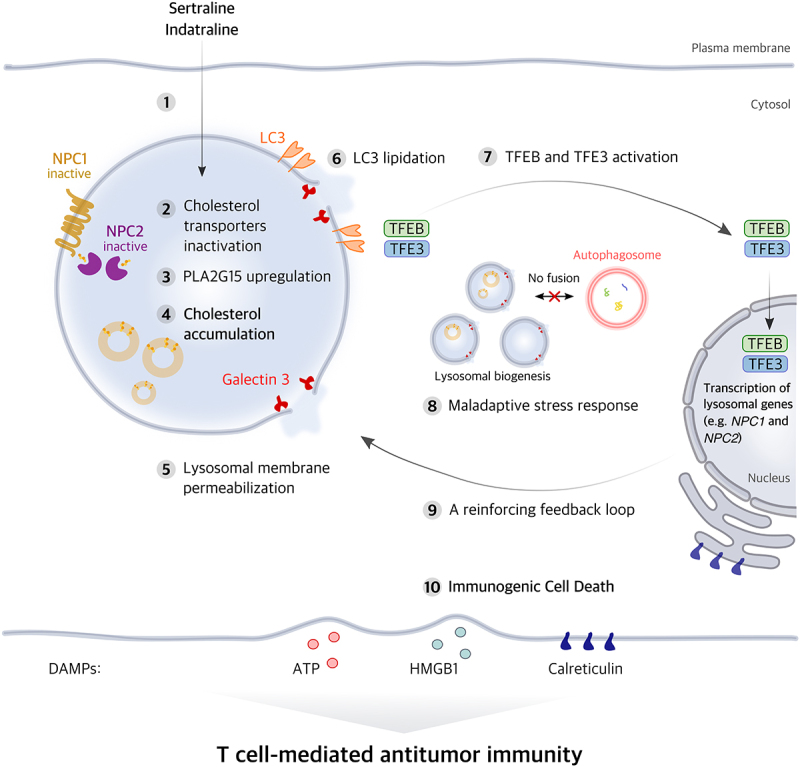
A proposed model for the induction of immunogenic cell death by the antidepressants, sertraline and indatraline.

Indatraline and sertraline exhibit the molecular characteristic of lysosomotropism, meaning that they tend to accumulate within lysosomes (1). Once inside the lysosome, they inactivate the lysosomal cholesterol transporters NPC1 and NPC2 (2) and trigger the upregulation of PLA2G15 (3). This leads to cholesterol accumulation within lysosomes (4) resulting in the permeabilization of the lysosomal membrane (LMP) (5) and disruption of autophagy. In addition, indatraline and sertraline induce TFEB activation through an LC3 lipidation-dependent but autophagy-independent mechanism (6 and 7). This triggers the increased expression of several lysosomal genes including *NPC1* and *NPC2*, as well as sustained biogenesis of lysosomes and autophagosomes. These TFEB/TFE3-dependent effects may initiate a reinforcing feedback loop that further enhance the accumulation of cholesterol and exacerbate lysosomal dysfunction (8 and 9). As a consequence, cancer cells undergo immunogenic cell death (ICD) characterized by the release of DAMPs (10). DAMPs, in turn, induce a T cell-mediated adaptive antitumor immune response and enhance the long-term efficacy of indatraline and sertraline.

DAMP, damage-associated molecular pattern; ICD, immunogenic cell death; LMP, lysosomal membrane permeabilization

Mechanistically, both compounds induced the accumulation of cholesterol in lysosomes, leading to LMP and subsequent ICD (Figure 1). Cholesterol depletion using β-cyclodextrin or low-density lipoprotein (LDL)-depleted medium abrogated LMP and the associated immunogenic features. Molecular docking analyses suggested that sertraline and indatraline may inhibit cholesterol binding to NPC1 and NPC2, critical lysosomal cholesterol transporters. Moreover, cells treated with these compounds exhibited multilamellar vesicles (MLVs), reminiscent of lysosomal storage disorders caused by NPC1 and NPC2 dysfunction,^10^ supporting this proposed mechanism. Interestingly, both compounds upregulated PLA2G15 (phospholipase A2 group XV), an enzyme that hydrolyzes the phospholipid bis(monoacylglycero)phosphate (BMP), present in lysosomal intraluminal vesicles.^11,12^

BMP plays a key role in regulating lysosomal lipid metabolism, particularly the efflux of cholesterol.^11,12^ Given the established link between defects in intracellular cholesterol transport with imbalances in sphingolipid and bis(monoacylglycero)phosphate levels in storage disorders, it is plausible that indatraline and sertraline may also disrupt broader lipid homeostasis.

Another layer of regulation involves TFEB and TFE3, master regulators of lysosomal genes.^13^ We demonstrated that both indatraline and sertraline activated TFEB and TFE3, resulting in upregulation of lysosomal genes, including *NPC1* and *NPC2*. Although TFEB and TFE3 activation is typically a pro-survival response to lysosomal stress,^14^ we speculate that, under these treatments, it constitutes a maladaptive feedback loop: upregulating lysosomal cholesterol transporters that are inhibited by both antidepressants, as well as promoting sustained biogenesis of lysosomes and autophagosomes. These responses exacerbate cholesterol accumulation and lysosomal damage, hence enhancing the cytotoxic and immunogenic effect of indatraline and sertraline (Figure 1).

It is important to highlight that perturbed intracellular cholesterol distribution could be due to an impairment of cholesterol transporters and trafficking in organelles other than lysosomes such as endoplasmic reticulum, mitochondria, and peroxisomes.^10^ Whether and how indatraline and sertraline affect global lipid metabolism and whether this regulation contributes to their pro-immunogenic effects warrants further investigation.

In addition to our findings in sarcoma models, evidence from murine models of melanoma and glioblastoma suggests that disruption of cholesterol homeostasis broadly suppresses tumor growth. Notably, several agents that impede cholesterol egress from lysosomes inhibit melanoma growth both *in* *vitro* and *in* *vivo*.^15^ Similarly, a recent study implicated the disruption of lysosomal cholesterol homeostasis by the paracaspase MALT1 (mucosa-associated lymphoid tissue lymphoma translocation protein 1) in glioblastoma suppression.^16^ It is worth noting that, unlike these previous studies, our research uniquely examined these effects in immunocompetent mice, explicitly assessing the immunostimulatory consequences of targeting lysosomal cholesterol transport. In contrast to these findings, a pro-tumoral role of cholesterol has been also reported suggesting that cholesterol plays a dual role in cancer depending on the cancer type and stage, the specific characteristic of cholesterol, and its precise distribution within intra- and extracellular compartments.^17,18^

Our findings establish a novel link between lysosomal cholesterol dysregulation and ICD, revealing a previously underappreciated mechanism of tumor immune activation. Along this line, the implication of lysosomal lipid peroxidation in ICD has been elegantly highlighted by Bhardwaj et al.^19^ who reported that DC661, a dimer of chloroquine that potently induces LMP, triggers ICD *via* PPT1 (Palmitoyl-protein thioesterase 1)-mediated ROS production and lysosomal lipid peroxidation. Whether lysosomal lipid peroxidation and cholesterol accumulation represent independent or interconnected pathways in LMP-driven ICD remains an open and intriguing question.

Dissecting the specific and shared features through which different LMP inducers regulate ICD is essential for optimizing this therapeutic strategy. A better understanding of these mechanisms may inform the rational development of combination treatments aimed at exploiting lysosomal vulnerabilities to enhance antitumor immunity.

## Data Availability

Data sharing is not applicable to this article as no new data were created or analyzed in this article. This article is a brief commentary on the article : Alvarez-Valadez, Karla et al. “Lysosomal damage due to cholesterol accumulation triggers immunogenic cell death.” *Autophagy* vol. 21,5 (2025): 934–956. doi: 10.1080/15548627.2024.2440842
